# Ancient Schwannoma of the Cervical Sympathetic Chain: A Rare Masquerader of Necrotic Cervical Lymphadenopathy

**DOI:** 10.7759/cureus.110569

**Published:** 2026-06-10

**Authors:** Fahad Idrees Shaikh, Vidisha Kubde, Kanchan Dhote, Snehal U Umate, Vivek Harkare, Nishka Tiwari

**Affiliations:** 1 Medicine, Narendra Kumar Prasadrao (NKP) Salve Institute of Medical Sciences and Research Centre, Nagpur, IND; 2 Otolaryngology - Head and Neck Surgery, Narendra Kumar Prasadrao (NKP) Salve Institute of Medical Sciences and Research Centre, Nagpur, IND

**Keywords:** ancient schwannoma, cervical sympathetic chain, horner’s syndrome, neck mass, necrotic lymphadenopathy

## Abstract

Ancient schwannoma is a rare, slow-growing, benign peripheral nerve sheath tumor characterized by long-standing degenerative changes that may clinically, radiologically, and histopathologically mimic malignancy. These tumors commonly present as asymptomatic, gradually enlarging masses in the head and neck region, posing significant diagnostic challenges. We report a rare case of cervical ancient schwannoma in a 53-year-old male who presented with a painless swelling below the left ear of six-month duration. Radiological imaging suggested a necrotic cervical lymph node, and fine-needle aspiration cytology was inconclusive due to hypocellularity. Complete surgical excision was performed, and definitive histopathological examination confirmed the diagnosis of ancient schwannoma. Postoperatively, the patient developed Horner’s syndrome, an expected but uncommon complication, which was managed conservatively. This case highlights the complex diagnostic challenges associated with ancient schwannomas, emphasizes the necessity of histopathological confirmation, and illustrates the importance of meticulous preoperative planning to anticipate potential neurological complications.

## Introduction

Schwannomas are benign, encapsulated nerve sheath tumors arising purely from Schwann cells [1]. While they are typically solitary and slow-growing, an estimated 25-40% of these lesions commonly occur in the head and neck region [2]. Within the cervical region, schwannomas most commonly originate from the vagus nerve, whereas tumors arising from the cervical sympathetic chain are considerably rarer [2]. Ancient schwannoma is a distinctly uncommon histological variant, a term first introduced to describe long-standing tumors exhibiting advanced degenerative changes, such as cystic necrosis, perivascular hyalinization, calcification, hemorrhage, and marked nuclear atypia [3]. These atypical features may mimic malignant peripheral nerve sheath tumors, creating diagnostic uncertainty [4].

Cervical ancient schwannomas, particularly those arising from the sympathetic chain, are quite rare and often present as asymptomatic, slow-growing neck masses [5]. Radiological findings frequently resemble necrotic lymphadenopathy, carotid body tumors, or malignant lesions [6]. Fine-needle aspiration cytology (FNAC) may remain inconclusive because of cellular degeneration and hypocellularity [7]. Complete surgical excision combined with detailed histopathological evaluation remains the gold standard for definitive diagnosis and therapeutic management [4]. We present a unique case of a cervical ancient schwannoma complicated by postoperative Horner’s syndrome following surgical excision, adding to the limited body of literature on this rare clinical entity [8].

## Case presentation

A 53-year-old male presented to the otorhinolaryngology outpatient department with a painless swelling below the left ear for six months. There was no history of fever, weight loss, dysphagia, dysphonia, respiratory difficulty, or neurological complaints. On examination, a firm, non-tender swelling measuring approximately 3×2 cm was noted in the left cervical region. The swelling was mobile and firm in consistency, and the overlying skin appeared normal. No local rise in temperature or cervical lymphadenopathy was observed (Figures 1A-1D).

**Figure 1:**
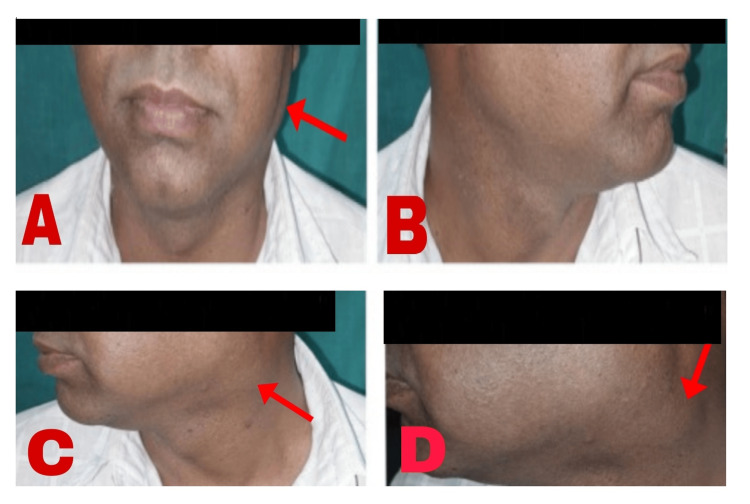
Preoperative clinical photographs of cervical schwannoma. (A) Frontal view showing subtle fullness of the left upper cervical region (red arrow) with preserved facial symmetry. (B) Right lateral view demonstrating a well-defined swelling along the lateral aspect of the neck. (C) Left lateral view showing the extent of the lesion (red arrow) without overlying skin changes. (D) Close-up lateral view highlighting the location and contour of the cervical swelling (red arrow), with normal overlying skin, suggestive of a benign, slow-growing nerve sheath tumor.

Contrast-enhanced computed tomography of the neck revealed a well-defined, heterogeneous, peripherally enhancing soft tissue lesion measuring approximately 2.6×4×4.3 cm, with a central non-enhancing necrotic area, in the left submandibular space, radiologically suggestive of a necrotic lymph node (Figures 2A, 2B).

**Figure 2:**
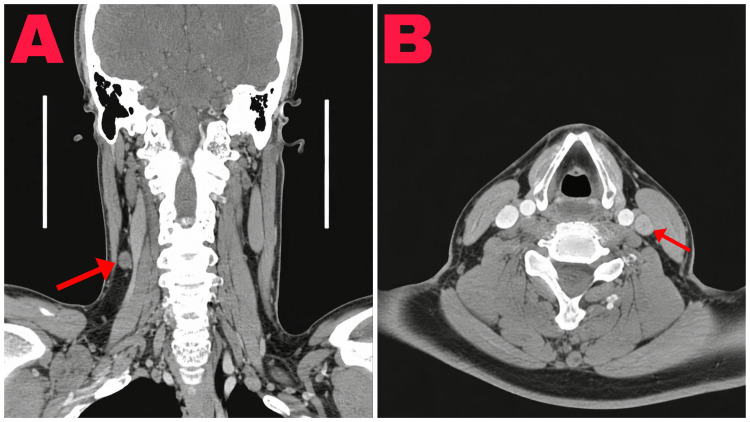
Contrast-enhanced computed tomography of cervical ancient schwannoma. (A) Contrast-enhanced CT scan of the neck (coronal view). Coronal section demonstrating a well-defined, heterogeneously enhancing soft tissue lesion in the lateral cervical region (arrow), located along the carotid space. The mass appears to displace adjacent vascular structures without evidence of invasion, consistent with a benign nerve sheath tumor such as cervical schwannoma. (B) Contrast-enhanced CT scan of the neck (axial view). Axial section demonstrating a well-circumscribed soft tissue lesion in the lateral cervical region (arrow), located within the carotid space. The lesion causes displacement of adjacent vascular structures without evidence of infiltration, a radiological feature suggestive of a benign nerve sheath tumor such as schwannoma.

Fine-needle aspiration cytology was inconclusive. The patient subsequently underwent complete surgical excision of the mass under general anesthesia. Intraoperatively, the lesion was found to occupy the carotid space and was meticulously dissected from the surrounding neurovascular structures and excised in toto (Figure 3).

**Figure 3:**
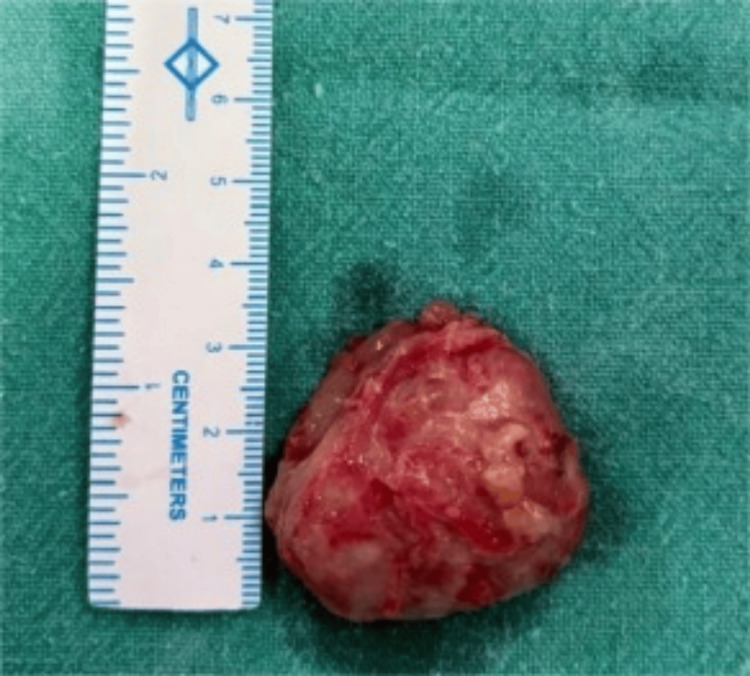
Excised specimen of cervical schwannoma. Gross specimen showing a well-encapsulated, ovoid soft tissue mass measuring approximately 3×4 cm (scale shown). The external surface appears smooth and lobulated with focal areas of congestion.

Histopathological examination revealed a spindle-cell neoplasm exhibiting the characteristic biphasic Antoni A and Antoni B architecture of schwannoma. In addition, prominent degenerative changes were identified, including nuclear pleomorphism and hyperchromasia in the absence of mitotic activity, features characteristic of ancient schwannoma. No evidence of malignant transformation was observed (Figures 4A, 4B).

**Figure 4:**
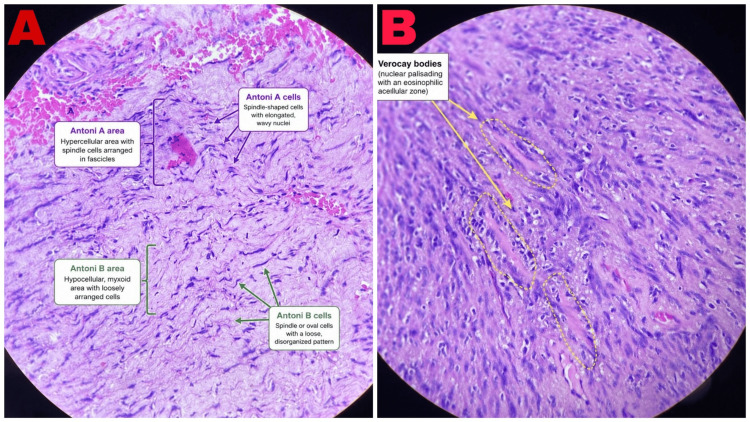
Histopathological examination of ancient schwannoma of the cervical region (H&E stain). (A) Photomicrograph showing characteristic biphasic architecture with hypercellular Antoni A areas composed of spindle-shaped cells with elongated wavy nuclei arranged in fascicles, along with hypocellular Antoni B areas demonstrating loosely arranged cells in a myxoid stroma (H&E stain, ×100 magnification). (B) Higher magnification demonstrating Verocay bodies formed by nuclear palisading surrounding eosinophilic acellular zones, consistent with schwannoma histopathology (H&E stain, ×400 magnification).

Postoperatively, the patient developed ipsilateral ptosis and miosis. Ophthalmological evaluation confirmed Horner’s syndrome; however, formal pharmacologic testing was not performed (Figures 5A-5C). Conservative management with regular follow-up was advised. The patient remained clinically stable during 12 months of follow-up, with no evidence of local recurrence.

**Figure 5:**
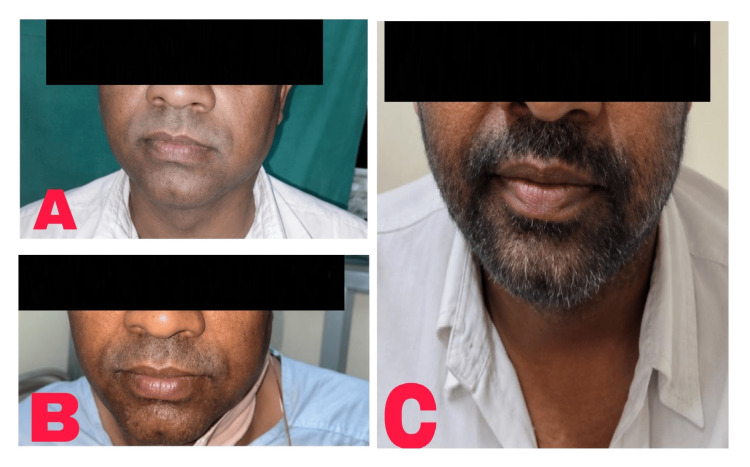
Postoperative clinical photographs following excision of cervical ancient schwannoma. Comparative images showing the preoperative appearance (A), immediate postoperative period (postoperative day one; B), and follow-up at postoperative day nine (C). The images demonstrate successful resolution of the cervical swelling following complete surgical excision. Mild ipsilateral ptosis, consistent with postoperative Horner’s syndrome, is evident during the postoperative period. No evidence of local wound-related complications or tumor recurrence is observed.

## Discussion

Schwannomas account for a significant proportion of benign peripheral nerve sheath tumors in the head and neck, but the “ancient” variant remains an exceptionally rare clinical entity [1,2]. The term "ancient neurilemmoma" was introduced by Ackerman and Taylor in 1951 to describe long-standing schwannomas exhibiting prominent degenerative changes, including hyalinization and nuclear atypia, in the absence of malignant behavior. The designation refers to the characteristic degenerative histopathological features rather than a specific anatomical location [3]. These atypical degenerative changes may closely mimic malignant peripheral nerve sheath tumors and other malignant cervical lesions, thereby creating substantial diagnostic uncertainty [4]. While the vagus nerve is the most commonly involved nerve in the neck, ancient schwannomas originating from the cervical sympathetic chain (CSC) are exceptionally scarce [5]. Only a limited number of cervical sympathetic chain ancient schwannomas have been reported in literature [6]. Our patient, a 53-year-old male, fits the typical demographic profile, as these tumors predominantly affect individuals in their middle-to-elderly years with no distinct sex predilection.

Clinically, ancient schwannomas are notoriously difficult to diagnose preoperatively because they typically present as solitary, slow-growing, painless neck masses, which was consistent with our patient’s six-month history of an asymptomatic swelling [7]. Because of their profound degenerative features, such as cystic necrosis and heterogeneous enhancement, radiological findings are frequently misleading [4]. In the present case, contrast-enhanced computed tomography (CT) suggested a necrotic lymph node. Similar diagnostic difficulty due to cystic degeneration and necrotic radiological appearance has been reported in recent literature [4]. Ancient schwannomas are frequently confused with cystic neck masses, branchial cleft cysts, or tubercular lymphadenopathy because of degenerative cystic areas [4]. However, subtle radiological clues can aid in differentiating the nerve of origin. In our patient, the lesion occupied the carotid space and displaced adjacent vascular structures, a hallmark of deep-seated cervical nerve sheath tumors. The apparent discrepancy between the clinically estimated size and the radiological dimensions likely reflects the deep location of the lesion within the carotid space. Clinical palpation may underestimate the true extent of deeply situated schwannomas, whereas cross-sectional imaging and pathological examination provide a more accurate assessment of tumor dimensions. Magnetic resonance imaging (MRI) is considered superior to CT for the characterization of schwannomas because of its excellent soft-tissue delineation and its ability to identify nerve sheath origin [4]. Although MRI is considered superior to CT for characterization of schwannomas and identification of the nerve of origin, MRI was not performed in the present case because the lesion was initially suspected to represent cervical lymphadenopathy, and CT findings were considered adequate for surgical planning. This represents a limitation of the preoperative evaluation.

The utility of fine-needle aspiration cytology (FNAC) for diagnosing ancient schwannomas is severely limited; in the present case, FNAC was inconclusive due to hypocellularity. This mirrors the broader literature; FNAC routinely yields a non-diagnostic, hypocellular, or hemorrhagic aspirate [7,4]. Consequently, definitive diagnosis invariably relies on postoperative histopathological examination [8]. Histologically, our specimen exhibited the classic biphasic architecture of hypercellular Antoni A areas and hypocellular Antoni B areas, alongside prominent nuclear pleomorphism and cystic degeneration. Immunohistochemical markers such as S-100 and SOX10 may further support the diagnosis of schwannoma and assist in excluding malignant peripheral nerve sheath tumors. However, immunohistochemical analysis was not performed in the present case because the diagnosis was established based on characteristic histopathological findings without evidence of malignancy. This should be considered a limitation of this study.

Complete surgical excision via an external approach remains the definitive treatment of choice because schwannomas are notoriously radioresistant tumors, while the prognosis is generally excellent and recurrence is exceedingly rare; surgical intervention in the carotid space carries an inherent risk of neurological morbidity [9]. Postoperative Horner’s syndrome, characterized by ptosis, miosis, and anhidrosis, is a widely recognized complication associated with cervical sympathetic chain schwannomas [10]. In the present case, the patient developed ipsilateral ptosis and a mid-dilated pupil postoperatively. Despite meticulous extracapsular dissection or enucleation, transient or permanent Horner’s syndrome may still occur because of unavoidable trauma or intricate sympathetic chain involvement within the tumor capsule.

Although malignant transformation of ancient schwannomas is phenomenally rare, isolated cases have been reported in literature [8]. This case underscores the necessity of maintaining a high index of suspicion for ancient schwannoma when evaluating complex cystic or necrotic cervical masses. It simultaneously emphasizes the indispensable role of meticulous histopathological evaluation in establishing a definitive diagnosis and highlights the importance of careful preoperative counseling regarding potential postoperative neurological deficits such as Horner’s syndrome.

## Conclusions

Ancient schwannoma of the cervical region is a rare, benign peripheral nerve sheath tumor that heavily mimics malignancy due to its advanced degenerative morphological features. Preoperative diagnosis remains a formidable challenge, as imaging frequently suggests necrotic lymphadenopathy or other malignant etiologies, and cytology is reliably inconclusive. The present case, together with the contemporary literature, confirms that histopathological examination is paramount for a definitive diagnosis. Complete surgical excision remains the gold standard treatment, offering an excellent prognosis. However, surgeons must anticipate and counsel patients on the significant risk of postoperative neurological complications, particularly Horner’s syndrome, when addressing tumors located near the cervical sympathetic chain.
